# Study of the Influence of Zirconium, Titanium and Strontium on the Properties and Microstructure of AlSi7Mg0.3Cu0.5 Alloy

**DOI:** 10.3390/ma15103709

**Published:** 2022-05-22

**Authors:** Dana Bolibruchová, Michal Kuriš, Marek Matejka, Justyna Kasińska

**Affiliations:** 1 Department of Technological Engineering, Faculty of Mechanical Engineering, University of Zilina, Univerzitná 8215/1, 010 26 Zilina, Slovakia; danka.bolibruchova@fstroj.uniza.sk (D.B.); michal.kuris@fstroj.uniza.sk (M.K.); 2 Department of Metal Science and Materials Technology, Kielce University of Technology, Al. Tysiąclecia Państwa Polskiego 7, 25 314 Kielce, Poland; kasinska@tu.kielce.pl

**Keywords:** Al-Si-Mg-Cu aluminum alloy, investment casting, zirconium, microstructure, mechanical properties

## Abstract

The aim of the paper is to describe and specify the properties and microstructure of Al-Si alloy using Zr, a combination of Zr with elements used in the grafting of Al (Ti) alloys, and modification (Sr). Al-Si alloys with a combination of Zr and Ti and Sr elements represent an opportunity for the development of new aluminum alloys with a specific use. The experiment focused on the analysis of the synergistic effects of Zr with Ti and Zr with Sr on a AlSi7Mg0.3Cu0.5 alloy. The experimental alloys contained a constant Zr content of 0.15 wt. % and were alloyed with a gradual addition of Ti and Sr in the range of 0.1 to 0.3 wt. % for Ti, and 0.1 to 0.3 wt. % for Sr. The experimental samples were cast by meltable model casting technology. In variants with a constant addition of Zr 0.15 wt. % and a gradual addition of Ti, we observed an increase in the values of mechanical characteristics, with a significant decrease in ductility. When evaluating the structure of experimental alloys, Ti affected it by increasing the number of precipitated Zr phases. Experimental alloys with Zr and Sr addition were characterized by nucleation of Zr phases in angular morphology. It can be concluded that the investigated elements are expected to have a positive (strengthening) effect even at higher operating temperatures.

## 1. Introduction

As the foundry and metallurgical industries are an integral part of industrial production, there is a need to make them more efficient while maintaining competitiveness on world markets [1]. There are several options, from improving the recycling of metal returnable material, through the modernization of production technologies, to the development of new materials that achieve a significant improvement in selected properties inherited from their predecessors [2,3,4]. 

A very important direction is the improvement of already used materials. At the same time, these components can be structurally lightened, because the functional cross-sections can withstand higher compressive or thermal loads during operation by increasing the strength characteristics (e.g., cylinder heads). The result is a reduction in the total weight and thus a saving in fuel or energy [5,6,7].

Current Al alloys use Cu to increase strength by hardening [8]. Commercially used hardenable Al-Si alloys are being replaced by new types of Al alloys that use either a system other than Al-Si (e.g., AlCu7MnZr) or are modified by alloying existing Al-Si alloys with selected elements (e.g., AlSi7MgCu0.5, AlSi8Cu3, or AlSi10Mg0.3Cu) [9,10,11]. 

The development of new, more sophisticated Al alloys can be oriented not only to change the content of the main alloying elements (e.g., Si, Mg, or Cu), but the alloys can also be influenced by a small number of specific alloying, inoculating, and modifying elements (such as Zr, Cr, Ni, Mo, Mn, V, Ti, and Sr, etc.) [12]. These are able to influence the crystallization of Al alloys, which significantly affects the change in selected properties. One of the possibilities is the application of Zr to Al alloys, which has an especially positive effect on the increase in strength, heat resistance, and heat strength (thermal strength) [13,14]. It is most often segregated in the form of intermetallic phases of Al_3_Zr or AlSiZr. Formation occurs during the peritectic reaction at Zr contents ≥ 0.1 wt. % [15].

The basic morphology of Zr intermetallic phases (dispersoids) is in the form of long acicular (needle-like) forms or plates, their size increasing with increasing Zr content. The individual needles are smooth with slightly split ends. The Zr phases are formed in two different crystallographic morphologies. The first is the DO_23_ tetragonal lattice, which is important for influencing the strength characteristics. However, the reduced symmetry of the tetragonal lattices causes an increase in brittleness, which has a negative effect on the ductility and modulus of elasticity. The L_12_ lattice is a cubic coherent lattice which, with its symmetry, compensates for the negative properties of the DO_23_ tetragonal lattice. Zr has the lowest diffusivity in Al alloys, while the individual Zr atoms are characterized by high binding energy. Al matrices containing intermetallic phases based on Al_3_Zr have increased resistance to coarsening and dissolution; it regulates the development of grains and subgrains and increases the strength and stability of Al matrix at higher temperatures (above 250 °C) [6,16,17,18,19]. 

In his work, Qian states that the addition of Zr obviously reduced the size of Si particles and changed its morphology. Grain size refinement was promoted by the reaction between Al and Zr, forming Al_3_Zr and ZrSi_2_ compounds. Compared to a Zr-free alloy, the additive enhanced the tensile strength, compressive strength, shear strength, and hardness [20]. On the other hand, Lu, in his work, describes how the addition of Zr significantly refined the microstructure of Al-Si-Cu-Mg baseline alloy, with the a-Al grain size decreasing from 335 to 253 µm and the SDAS decreasing from 39 to 28 µm. The microstructure refinement resulted from the solute segregation effect and the heterogeneous nucleation via pro-peritectic Zr-containing phase, serving as the effective nucleant particles. Meanwhile, the measured maximum size of porosity was largely decreased from 368.5 to 276.4 µm (by 25%) [21].

The effect of Zr in Al alloys can be multiplied via combination with other elements. These complicated systems are currently not sufficiently explored.

As is generally known, Ti is used in Al alloys as an inoculating agent, which allows the formation of crystallization nuclei and refines the structure of the α phase. The addition of Zr and Ti to Al-Si alloys results in a more intensive grain refinement compared to using the individual elements alone. Ti is formed in the form of the Al_3_Ti phase, which is characterized by the DO_22_ tetragonal lattice. The atomic distance in the DO_22_ lattice from the nearest neighbor is around 0.2826 nm in the {112} plane, while it is 0.2856 nm in the {111} plane. The low permeability of the crystallographic lattice in the respective contact planes indicates an increasing inoculating effect in Al alloys. The synergistic effect of Zr and Ti should increase the strength properties and hardness, but also probably the heat resistance of Al-Si alloys. However, the average interatomic distance in the {114} plane affects a significant reduction in the inoculating effect when Zr and Ti are applied separately [5,19,22]. Zirconium reacts Ti, Si, and Al to form the phases (Al,Si)_2_(Zr,Ti) and (Al,Si)_3_(Zr,Ti). The Zr-rich intermetallic phases appear in two different forms: the phase (Al,Si)_2_(Zr,Ti) containing high levels of silicon which is block-like in form, and the phase (Al,Si)_3_(Zr,Ti) containing high levels of aluminum which appears in needle-like form [23]. The interaction of Zr with Ti in Al-Si alloys can also help in the formation of crystallization nuclei of Zr phases, strengthen the refinement of grains and subgrains, and improve selected mechanical properties [24,25]. In practice, however, boron is also used along with Ti, which greatly improves the grain refinement ability. Boron together with Ti and Al form intermetallic phases such as AlB_2_, TiB_2_, or (Al, Ti)B_2_ with a size of 0.5 to 2 μm, which are much smaller particles than TiAl_3_. These TiB_2_ particles also serve as heterogeneous nuclei [26,27].

Sr is added to Al alloys as a modifier of eutectic Si, which affects the change in Si morphology from coarse plates to finer rods. It has the most effective modifying effect in molds with rapid cooling. Due to the “absorption” of Sr by the intermetallic phases of other metals, a change occurs. The synergistic action of Zr and Sr can have a positive effect on the reduction in grain size, and this effect depends on their weight ratios in the alloy. Sr, together with Zr, forms Al_3_(Sr1-x Zrx)-based phases that can act as nucleating nuclei. The increased content of Sr can cause the formation of SrO and Al_2_SrO_3_ oxides, which have a negative effect on the melt [6,13,23,24]. However, research has shown that the presence of small-size oxides helps to form nucleation site, especially those based on Al_3_Zr. Unlike the combination of Zr and Sc or Zr and Ti, the combination of Zr and Sr in aluminum alloys does not change the structure to non-dendritic and, at the same time, the given phases can bind Fe to them [28,29,30,31,32,33,34]. 

The aim of the paper is to verify the synergistic effect of Zr with Ti and Zr with Sr on the AlSi7Mg0.3Cu0.5 alloy products made by investment casting technology, because the issue of improving properties by adding Zr, especially in combination with Ti or Sr, is very rarely investigated in the given research area. The area of interest is focused on gaining knowledge in the development of a more sophisticated hardenable alloy, which is based on the AlSi7Mg0.3Cu0.5 alloy. The main emphasis is placed on obtaining better performance properties of the newly formed alloy. The results of influencing the investigated alloy will be able to focus on the possibility of improvement in mechanical properties and lightening automotive components and parts without the risk of weakening.

## 2. Materials and Methods

The experiments were divided into two material variants, which differ in the content of Ti and Sr. Both Ti and Sr were introduced into the melt with the co-acting Zr. The results were compared with an alloy without the addition of the alloying element (AlSi7Mg0.3Cu0.5 = R alloy) and with an alloy with a constant amount of Zr 0.15 wt. % (AlSi7Mg0.3Cu0.5Zr0.15 = P alloy).

Experimental work was performed at three levels of alloying elements contents for the Z + Ti and Zr + Sr material variants. The newly formed material variants were compared with alloys without the addition of alloying elements (R alloy) and with the addition of only Zr (P-alloy) (Table 1). To simplify the marking, only the letters of the experimental alloys (listed in Table 1) are used in the other parts of the paper, and not the chemical marking (formulas) of the alloys. The chemical composition was determined by arc spark spectroscopy (Bunker—Q2 ION, Kalkar, Germany). 

**Figure 1:**
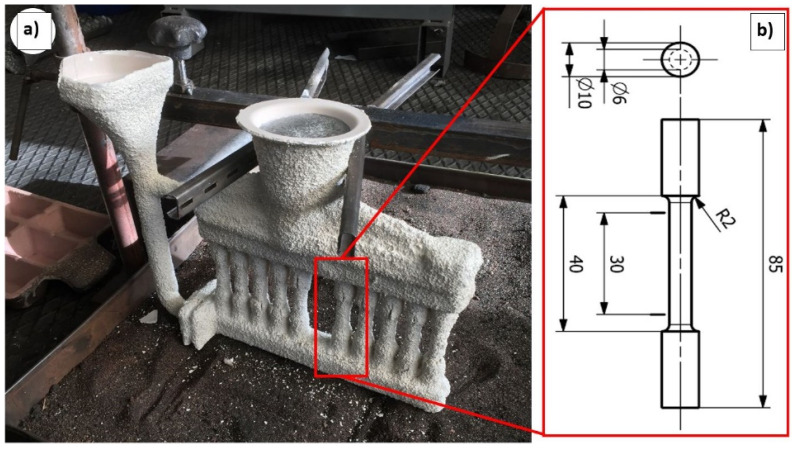
(**a**) Ceramic mold supplied by AluCAST s.r.o., (**b**) Scheme of test specimen.

The individual material variants were cast into ceramic molds by investment casting technology (Figure 1). The ceramic mold was made in three layers (contact, insulating, and reinforcing), and the material characteristics are given in Table 2. The ceramic molds were fired at a temperature of 750 ± 10 °C for 1.5 h. Subsequently, the ceramic molds were removed from the furnace and prepared for casting. The ceramic molds had a surface temperature of 510–540 °C before casting. The casting temperature of the experimental alloys was 750 ± 10 °C. The casting height reached 200 mm from the inlet well. The casting velocity was 0.6 kg·s^−1^. After casting, the ceramic mold was cooled in air for 1 h. The AlSi7Mg0.3Cu0.5 alloy is not standardized by the EN 1706 standard and has application possibilities, especially in the automotive industry. The AlSi7Mg0.3Cu0.5 alloy was already delivered by the manufacturer in a pre-modified and pre-inoculated state. The melts were intentionally not degassed.

Zr was added in the form of AlZr15 master alloy, Ti in the form of AlTi5B master alloy, and Sr in the form of AlSr10. From each material variant, half of the samples were heat treated (HT) by precipitation hardening T6 (5 pieces out of 10 pieces of cast samples from one experimental melt). The T6 heat treatment regime consisted of solution treatment (540 ± 5 °C/12 h), quenching (water 66 ± 2 °C), and artificial aging (155 ± 5 °C/5 h). Experimental alloys E1 to E3 were alloys with a graded addition of Ti in the range from 0.1 wt. % to 0.3 wt. % Ti. Experimental alloys F1 to F3 were modified with a graded amount of Sr in the range from 0.1 wt. % to 0.3 wt. %.

The evaluation of the density index was performed through the so-called density index (DI). The molten test alloy was poured into two 60 mL test crucibles. The principle of the method is to compare the density of a sample of an alloy which solidified at atmospheric pressure with a sample which solidified in a vacuum chamber (0.097 MPa, for 4 min). By comparing the weights of the samples in air and water, the density was calculated, and the density index was determined according to a simple equation:

(1) $$\mathrm{DI}=\frac{\rho_{\mathrm{atmosphere}}-{\;\rho }_{\mathrm{vacuum}}}{\rho_{\mathrm{atmosphere}}}\;\times \;100\;\left[\%\right]$$

where ρ_atmosphere_ indicates the density of the sample solidifying freely at atmospheric pressure (g·cm^−3^) and ρ_vacuum_ is the density of the sample solidifying at near-vacuum (g·cm^−3^).

The process of crystallization of the alloys was evaluated by thermal analysis. A K-type (NiCr-Ni) thermocouple placed in the center of a cylindrical metal mold with a diameter of 34 mm and a height of 50 mm was used during the measurement. Values were recorded in LabView 2 Hz software (version 18.5, National Instruments, Austin, TX, USA). LabView software recorded changes in alloy temperature over time in a file. Based on the exact values of temperatures and time, specific values of temperatures for individual structural components of all observed alloys were determined [35,36]. 

Mechanical tests were performed on an Inspekt Table 50 kN rupturing device according to EN ISO 6892-1. All reported values of mechanical characteristics are average values from 5 measurements. Samples that reached the highest values of mechanical characteristics were selected to evaluate the microstructure after heat treatment. Samples were prepared by standard procedures, but to increase Zr-rich phase contrast, samples were etched with H_2_SO_4_. Samples were evaluated using an optical microscope (OM) Nikon Epiphot 200 (Nikon, Tokyo, Japan) and scanning electron microscope (SEM) observations with energy-dispersive detectors Oxford Ultim Max scanning electron microscope 65 (Oxford Instruments, Abingdon, UK).

## 3. Results 

### 3.1. Results of Thermal Analysis

**Figure 2:**
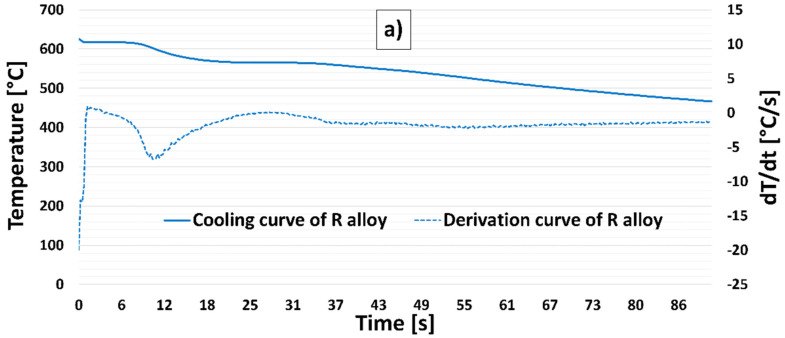

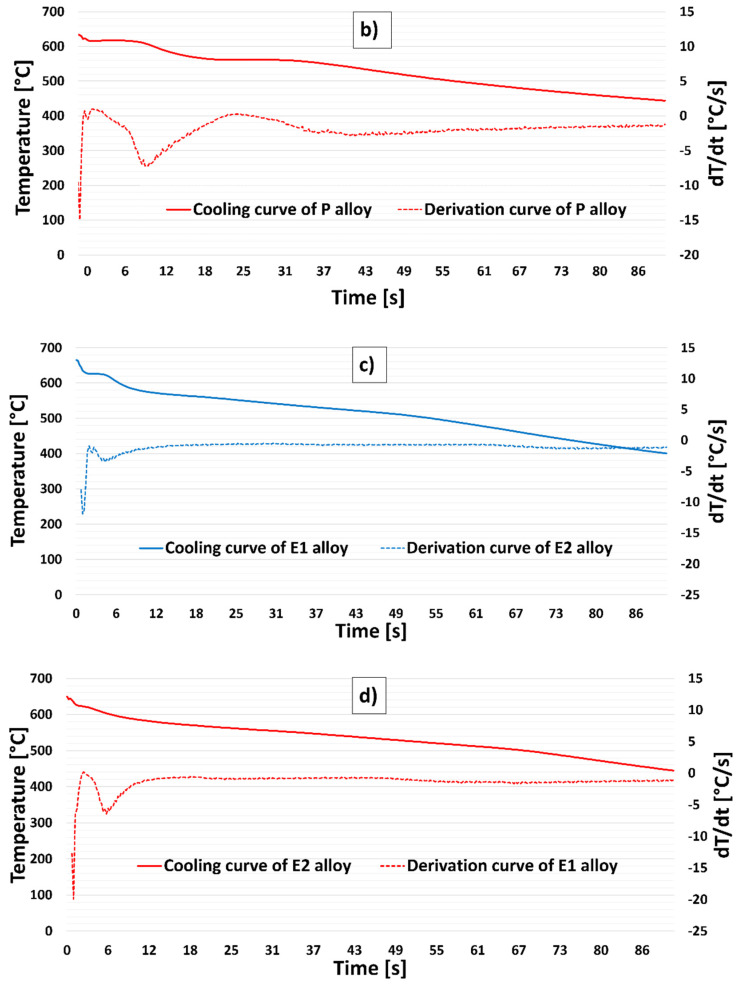

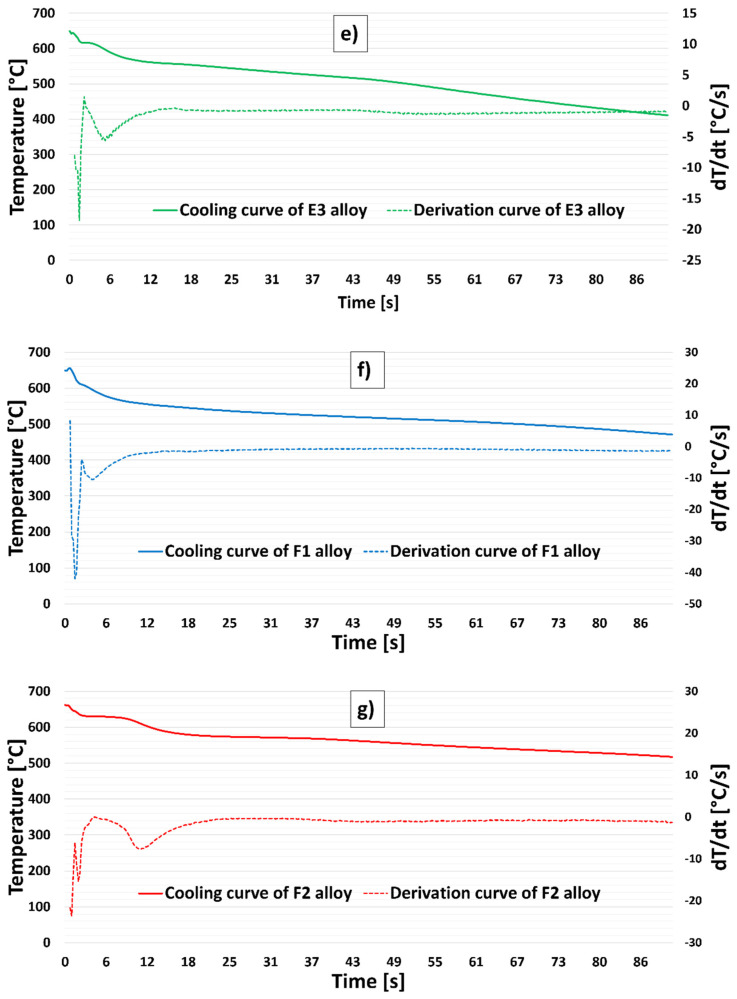

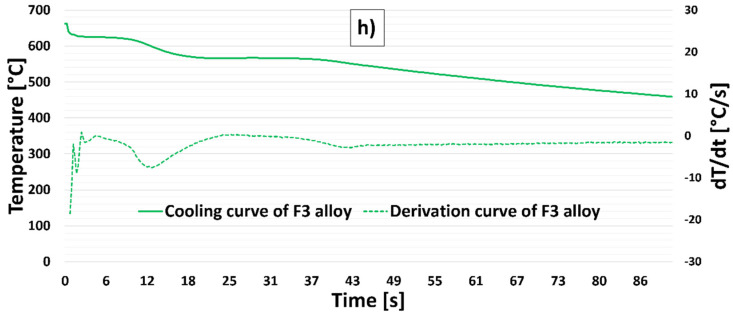
Cooling curves of their first derivative of experimental alloys; (**a**) R alloy, (**b**) P alloy, (**c**) E1 alloy, (**d**) E2 alloy, (**e**) E3 alloy, (**f**) F1 alloy, (**g**) F2 alloy, and (**h**) F3 alloy.

Thermal analysis was used to analyze the influence of the synergistic effect of Zr with Ti and Zr with Sr on the crystallization process of alloys (E1–E3 and F1–F3, respectively) and compared with the alloy R and alloy P (with constant Zr content). The aim of the thermal analysis was to identify the range of segregation temperatures of new components (intermetallic phases, dispersoids, etc.) and to find their influence on the AlSi7Mg0.3Cu0.5 alloys solidification process. Figure 2 shows the cooling curves and their first derivatives for all experimental alloys.

When evaluating the thermal analysis of alloy R and alloy P, only minimal differences in the nucleation temperatures of the structural components (α phase, eutectic, and intermetallic phases) can be observed (Figure 2a). A noticeable difference occurred due to the crystallization of the Zr phases in alloy P (with 0.15 wt. % Zr). The Zr phases in alloy P were precipitated at 630 °C, thus confirming the results of our previous work, where the peak of the curve in the temperature range 610 °C to 630 °C was repeatedly recorded as the Zr content in the investigated alloy increased [37].

In the case of the combination of Zr and Ti (E1 to E3 alloys), we observed an increase in the Zr-rich phase precipitation temperature range to 640 °C to 645 °C (Figure 2b and Table 3) compared to alloy P. No significant difference in α phase precipitation temperature and eutectics was observed for E1–E3 alloys (Table 3) compared to R and P alloys, but a difference in the shape of the cooling curves and their first derivatives was recorded. 

For alloys F1 to F3 (combination Zr and Sr), the precipitation of Zr phases (from the original 630 °C for the P alloy) increased slightly to approximately 633 °C to 636 °C (Figure 2c and Table 4). Significant changes in the elimination of structural components in alloys F1 to F3 were observed in eutectic elimination (Table 4). 

### 3.2. Evaluation of Density Index

The formation of gas pockets (bubbles) is closely related to the presence of foreign nuclei. DI expresses the cumulative effect of the content of gases and oxide inclusions, i.e., the true tendency of the alloy to form gas pockets and to gasify. 

**Figure 3:**
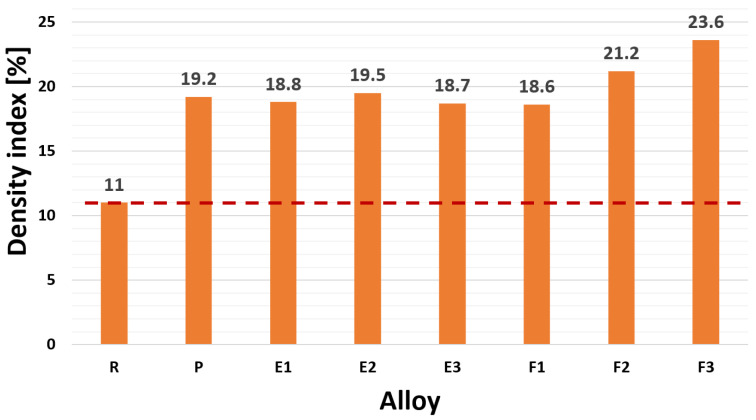
Relationship between density index and experimental alloys R, P, E1–E3, and F1–F3.

Due to the intentional absence of degassing, it was possible to observe a significant increase in the density index with increasing content of Zr (alloy P), Zr with Ti (alloys E1–E3), and Zr with Sr (alloys F1–F3) in the AlSi7Mg0.3Cu0.5 alloy (Figure 3). Compared to alloy R (reference value—red line in Figure 3), an increase in DI index of up to 73% is observed for alloy P. The value of DI index in the material variant Zr with Ti was at approximately the same level as in alloy P (DI = 18.7% to 19.5% of alloy E1–E3). The material variant Zr with Sr (alloys F1–F3) was characterized by the most significant increase in the DI index. Increasing wt. % Sr in the alloy increased the value of DI, while the highest value DI was measured in the alloy F3 (DI = 23.6%). 

### 3.3. Evaluation of Microstructure, Fracture Surfaces and EDX Phase Analysis

**Figure 4:**
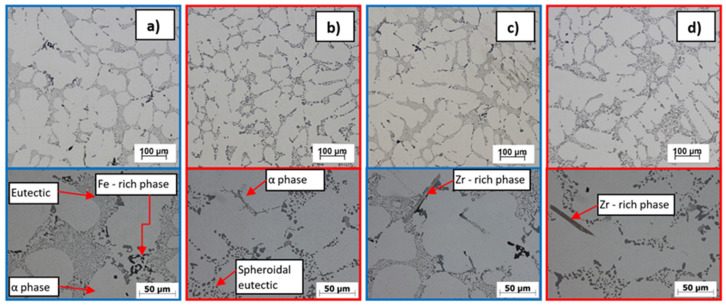
Microstructure of experimental alloys, H_2_SO_4_ etch.; (**a**) R—AlSi7Mg0.3Cu0.5 alloy in cast state, (**b**) R—AlSi7Mg0.3Cu0.5 alloy after heat treatment, (**c**) P—AlSi7Mg0.3Cu0.5Zr0.15 alloy in cast state, and (**d**) P—alloy AlSi7Mg0.3Cu0.5Zr0.15 after heat treatment.

**Figure 5:**
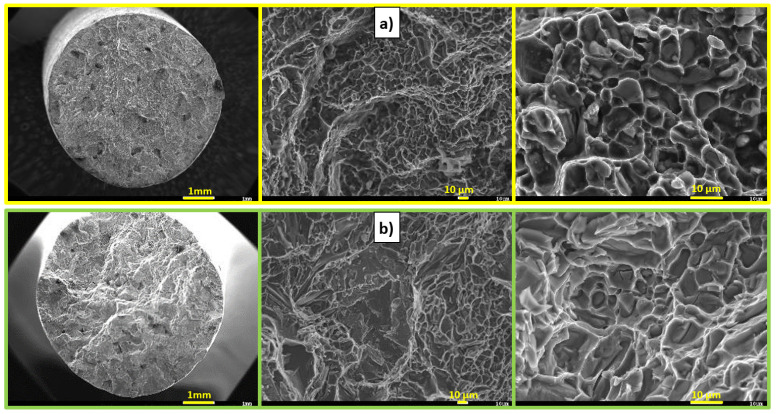
Macroscopic and microscopic character of fracture surfaces after heat treatment, SEM; (**a**) R alloy, (**b**) P alloy.

AlSi7Mg0.3Cu0.5 (R alloy—Figure 4a,b) is a pre-inoculated, pre-modified sub-eutectic alloy with a modified eutectic and refined α phase dendrites. The phases of Al_2_Cu and Mg_2_Si were also observed in the structure, which enable the hardening of the alloy. The harmful content of Fe was compensated by a suitable content of Mn and therefore it is possible to observe the Fe-rich phases in the form of so-called Chinese script. Visible formation of Zr phases in the AlSi7Mg0.3Cu0.5Zr0.1 metal matrix (P alloy) occurs at Zr content ≥ 0.1 wt. % already (Figure 4c,d). Zr is most often formed in the form of long smooth needles with slightly split ends, with Zr content occurrence expected increase in Zr phases. These phases disintegrated due to heat treatment, and the given disintegration was more significant when the content of Zr was ≥0.2 wt. % in the alloy (Figure 5d). This fact indicates a reduced degree of stability of the thus formed Zr phases in the P alloy.

The R alloy matrix (Figure 5a) is formed by a solid α solution with a K12 lattice, which is characterized by high plasticity. Intermetallic phases are formed in the metal matrix of the α phase. During the disruption of the matrix, only ductile failure occurs, but the presence of brittle phases in the structure causes the occurrence of fissile facets on the fracture surface. Due to heat treatment, the modified eutectic in the form of fibers changes to spherical. The result is a transcrystalline ductile disruption of the matrix with pit-shaped morphology and plastically deformed ridges of α-phase dendrites. In the P alloy matrix of AlSi7Mg0.3Cu0.5Zr0.1 (Figure 5b), it is possible to observe a decrease in the ductile disruption of the matrix due to the formation of an increased number of Zr phases. At the same time, it is possible to observe the occurrence of larger fission facets on the fracture surfaces of the alloy. 

**Figure 6:**
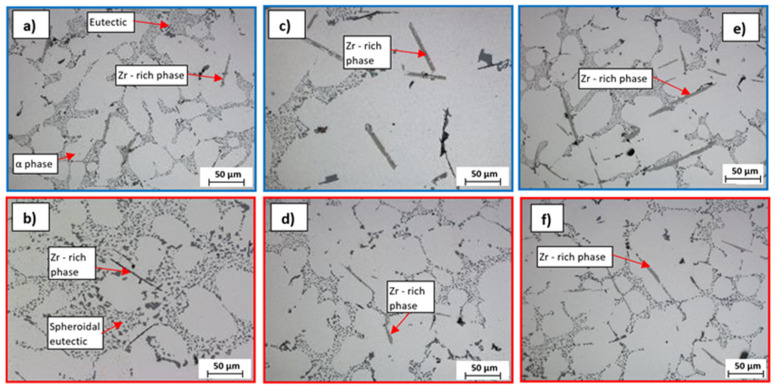
Microstructure of experimental alloys, H_2_SO_4_ etch., (**a**) alloy E1 0.1 wt. % Ti in the cast state, (**b**) alloy E1 0.1 wt. % Ti after heat treatment, (**c**) alloy E2 0.2 wt. % Ti in the cast state, (**d**) alloy E2 0.2 wt. % Ti after heat treatment, (**e**) alloy E3 0.3 wt. % Ti in the cast state, and (**f**) alloy E3 0.3 wt. % Ti after heat treatment.

In the case of Zr with Ti alloys (alloy E1–E3), an increased number of Zr phases (Figure 6a,c,e) were formed. These were characterized by a more coherent, shorter morphology without split ends. The increase in the number of Zr phases was almost twofold in the given material variants compared to alloys with only the addition of Zr. At the same time, a uniform ratio of intermetallic Zr phases with a longer sharp-edged and acicular morphology and a more integral angular morphology was observed. In the alloys E1 to E3, along with Ti, B was also added to the melt via the AlTi5B master alloy. The TiB_2_ phase most often occurs in hexagonal morphology with a particle size of 0.5 to 2 μm. However, in alloys E1 to E3, the TiB_2_ phase was not observed in the plane of the image from the optical and scanning electron microscope.

**Figure 7:**
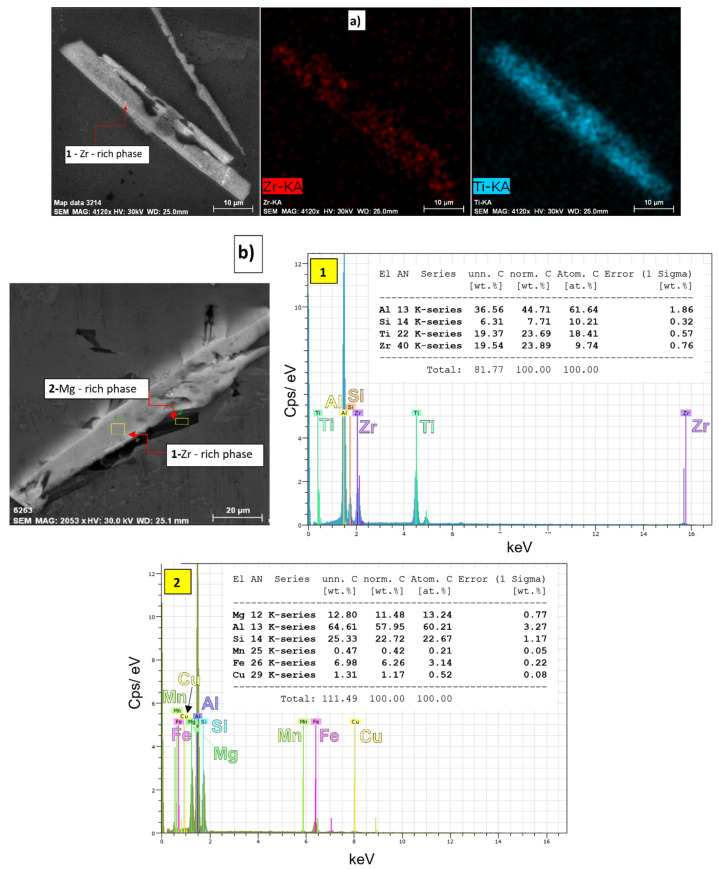
Identification of Zr-rich phases in the primary alloy P, SEM; (**a**) Mapping, (**b**) EDX analysis.

Intermetallic phases of the Al_3_Zr and AlSiZr types were observed by EDX and mapping analysis (Figure 7). Vončina also identifies a similar morphology of Zr phases in his work [16]. EDX analysis shows that in the Zr-rich phase (needle morphology) Si is present and especially in larger content of Ti (Figure 7b). The analysis also shows that, in the vicinity of the Zr-rich needle phase, Mg-rich phases with a smaller proportion of Fe and Cu crystallize. Mapping in Figure 7a shows the distribution of Zr and Ti in the aluminum matrix and Zr-rich phase (needle morphology).

**Figure 8:**
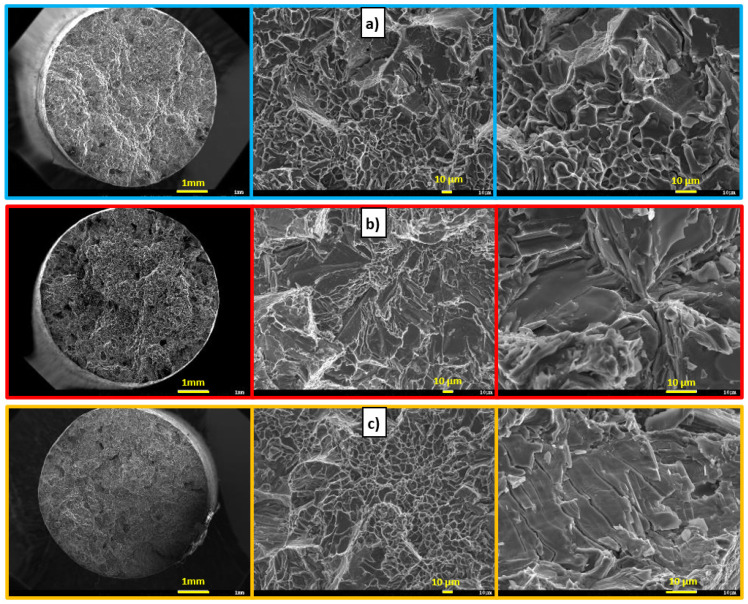
Macroscopic and microscopic character of fracture surfaces after heat treatment, SEM; (**a**) E1 alloy, (**b**) E2 alloy, and (**c**) E3 alloy.

In contrast to the P alloy, there was no significant disintegration of Zr phases (Figure 6b,d,f) after heat treatment. It can be concluded that finer, evenly distributed intermetallic Zr phases have a reinforcing effect in the AlSi7Mg0.3Cu0.5 alloy. In the case of experimental alloys E1 to E3 (Figure 8), it is possible to observe a decrease in ductile matrix disruption due to the precipitation of a much larger number of Zr phases than in the case of the P alloy. Although the size of the fission facets is slightly smaller than in the case of the P alloy, it is possible to observe an increase in their number. Therefore, the mechanism of transcrystalline ductile failure was applied in the formation of the fracture surface.

**Figure 9:**
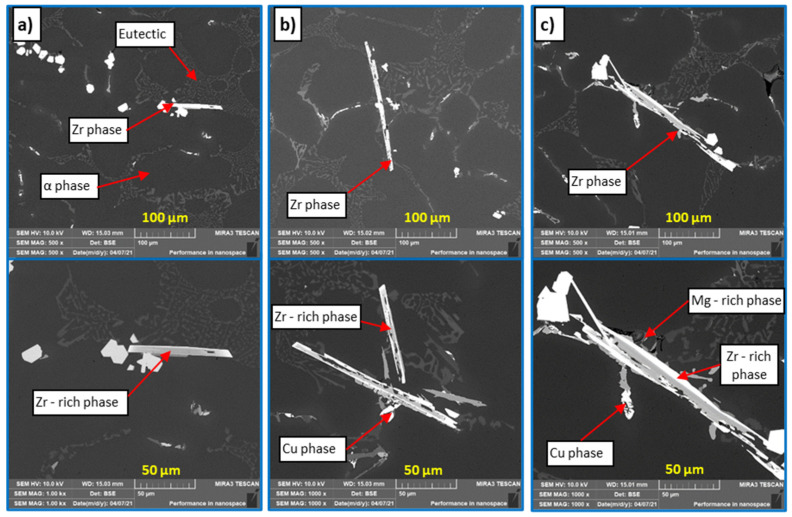
Microstructure of experimental alloys, H_2_SO_4_ etch., (**a**) alloy F1 0.1 wt. % Ti in cast state, (**b**) alloy F2 0.2 wt. % Ti in cast state, and (**c**) alloy F3 0.3 wt. % Ti in cast state.

**Figure 10:**
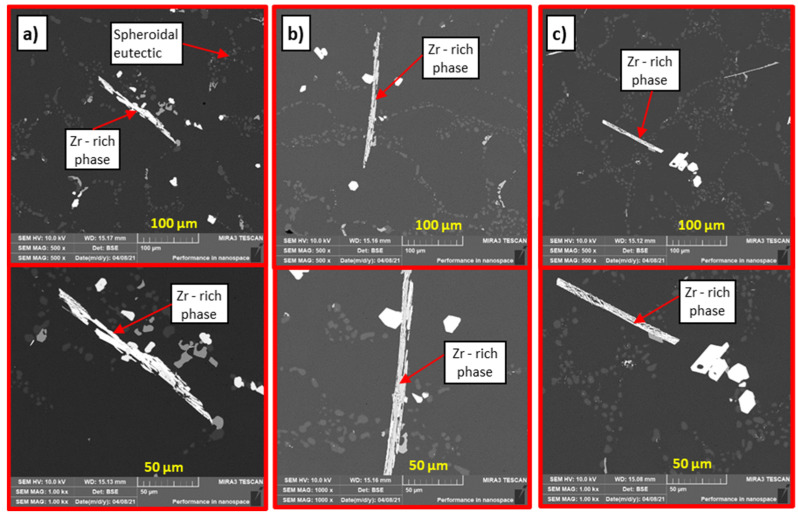
Microstructure of experimental alloys, H_2_SO_4_ etch., (**a**) alloy F1 0.1 wt. % Ti after heat treatment, (**b**) alloy F2 0.2 wt. % Ti after heat treatment, and (**c**) alloy F3 0.3 wt. % Ti after heat treatment.

Experimental alloys F1 to F3 were characterized by high porosity in the observed areas. The effect of the Zr and Sr combination on the alloy gas content was analyzed by the SEM method. When observing the structure of alloys F1 to F3, in contrast to alloys E1 to E3, a lower number of formed Zr phases was observed with a size as in the case of the P alloy (Figure 9 and Figure 10). The difference presented in the form of Zr phases, which had a predominantly angular shape. The formed Zr intermetallic phases interacted with Mg- and Cu-based phases. Zr phases were observed mainly in the eutectic domain. With increasing Sr content in alloys F2 to F3, a shortening of the formed Zr phases and a change in morphology to angular was observed. The formation of Zr phases in the eutectic region, together with Mg- and Cu-based phases, was also identified (Figure 9). Due to heat treatment, the Zr phases disintegrated (Figure 10) in contrast to alloys with Zr and Ti. 

**Figure 11:**
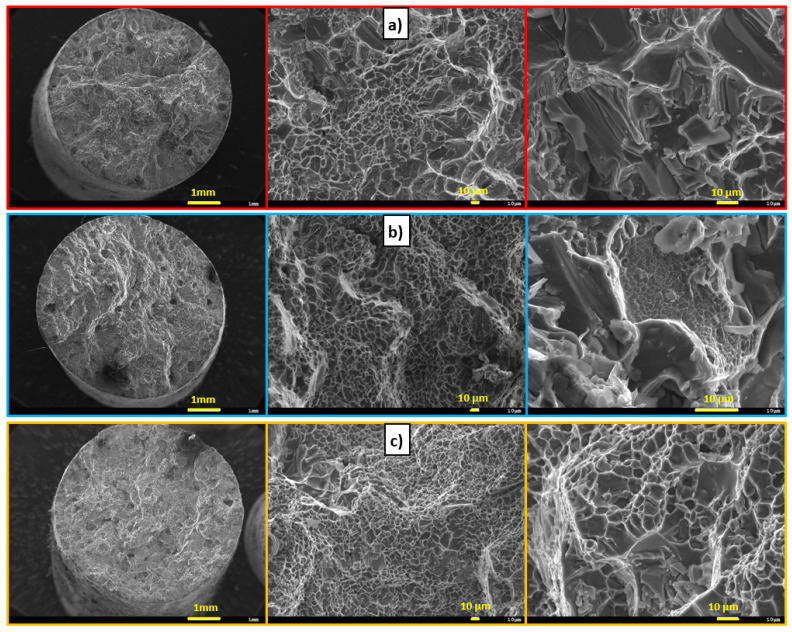
Macroscopic and microscopic character of fracture surfaces after heat treatment, SEM, (**a**) F1 alloy, (**b**) F2 alloy, and (**c**) F3 alloy.

In contrast to experimental alloys E1 to E3, it is possible to observe a slight decrease in ductile matrix disruption due to the formation of much larger Zr phases (similar to the P alloy). The mechanism of transcrystalline ductile disruption is applied in the formation of the fracture surface (Figure 11b).

### 3.4. Mechanical Properties

#### 3.4.1. Tensile Strength R_m_ and Yield Strength R_p0.2_

**Figure 12:**
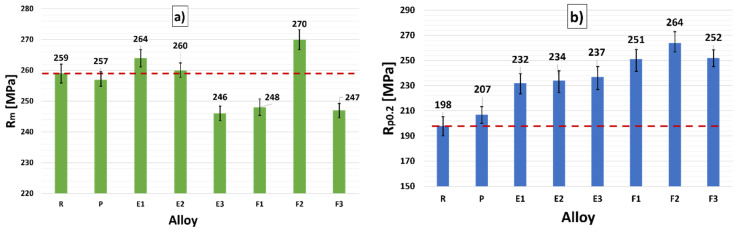
Dependence of (**a**) R_m_ and (**b**) R_p0.2_ after heat treatment for R, P, and experimental alloys E1–E3 and F1–F3.

In the evaluation of mechanical characteristics, material variants with addition of Zr with Ti (alloys E1–E3) and Zr with Sr (alloys F1–F3) were compared with the R alloy (without addition of alloying elements) and the P alloy (only with 0.15 wt. % Zr). The average values for tensile strength and yield strength, as well as respective standard deviations, are presented in Figure 12. The mechanical properties of the R alloy provided the so-called reference parameters (red line in Figure 12), i.e., these are the characteristics of the alloy without added alloying elements. Alloy is not standardized, so they were taken as a “baseline”.

The P alloy achieved a decrease of only 0.9% R_m_ compared to the R alloy. The R_p0.2_ value increased by 4%. Experimental samples E1 to E3 represented a variant where the synergistic effect of Zr with Ti increased the mechanical characteristics even at a lower Zr content. When evaluating alloys E1 to E3, there was an increase in R_m_ compared to R alloy by about 2%. The increase occurred in the E1 alloy. On the contrary, a decrease in R_m_ was recorded for alloy E3 (about 5%) compared to the R alloy. The most significant increase in mechanical characteristics in samples E1 to E3 was recorded at R_p0.2_. The increase compared to the R alloy was by about 20%, and compared to the P alloy, about 15%. The highest increase was recorded for sample E3. 

When evaluating the mechanical properties of samples F1 to F3, a slightly higher decrease in the monitored values was observed compared to samples E1 to E3. Regarding R_m_, there was a decrease of 5% compared to the R and P alloys. An increase was observed in sample F2 (by about 4% compared to R alloy and by about 5% compared to P alloy). The increase compared to alloy E1 (AlSi7Mg0.3Cu0.5Zr0.15Ti0.1), evaluated by us as the best, was only by about 2%. When evaluating R_p0.2_ in alloys F1 to F3, there was an increase in values, while alloys F1 to F3 also reached higher values than alloys of variant E. The alloy with the highest value was F2 (AlSi7Mg0.3Cu0.5Zr0.15Sr0.2), with an increase of 33% compared to the R alloy and 27% compared to the P alloy.

#### 3.4.2. Ductility A_5_ and Modulus of Elasticity E

**Figure 13:**
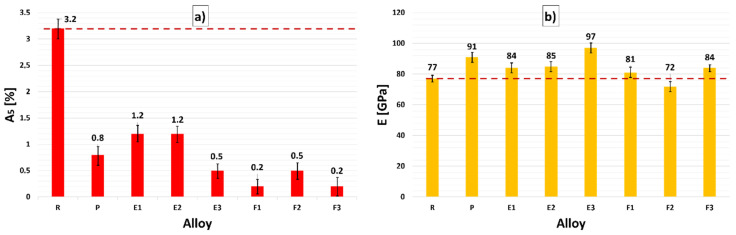
Dependence of (**a**) elongation A and (**b**) modulus of elasticity E after heat treatment for R, P, and experimental alloys E1–E3 and F1–F3.

The average values for ductility and modulus of elasticity of experimental alloys, as well as respective standard deviations, are presented in Figure 13. In the analysis of the values of ductility A_5_, a decrease was observed for the P alloy to a higher extent compared to the evaluation of R alloy. There was a decrease by almost 75% compared to the R alloy. 

The modulus of elasticity of the alloy P (with the addition of 0.15 wt. % Zr) increased by 19%. The modulus of elasticity was the parameter with which the largest increase in values occurred. 

When evaluating samples E1 to E3 (alloy with addition of Zr with Ti), a decrease in ductility A_5_ similar to the P alloy was recorded in all samples by 61% to 84% compared to the R alloy, and by 37% to 55% compared to the P alloy. The most significant decrease occurred in sample E3, in which a uniform ratio between the formed phases with sharp edges and more integral angular Zr phases was observed on the microstructure (Figure 7e). When evaluating the modulus of elasticity, a smaller increase was observed for samples E1 and E2 compared to the R alloy. In the case of the P alloy, the values decreased by approximately 8%. The increase in modulus of elasticity was recorded only in sample E3.

As with samples E1 to E3, samples F1 to F3 showed a significant decrease in the ductility of A_5_ by up to 85% to 95% relative to the R alloy and a 42% to 78 % decrease relative to the P alloy. For the P alloy, there was a decrease in A_5_ in all samples in the range of 7% to 20%.

The modulus of elasticity manifested the only significant increase in the case of the E3 alloy. 

#### 3.4.3. HBW Hardness

**Figure 14:**
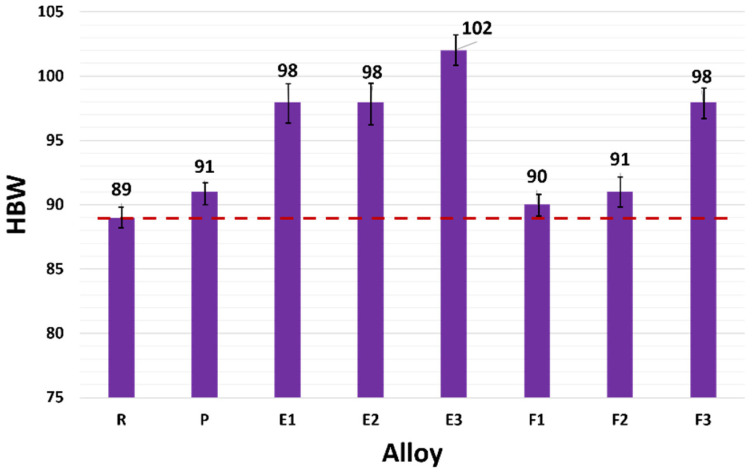
Dependence of HBW after heat treatment for R, P, and experimental alloys E1–E3 and F1–F3.

The average values for HBW hardness of experimental alloys, as well as respective standard deviations, are presented in Figure 14. When evaluating the Brinell hardness, an increase in the values of P alloy by 3% compared to R alloy (reference value—red line in Figure 14) was observed. The HBW hardness of samples E1 to E3 increased by about 15% compared to R alloy. The increase in HBW hardness compared to P alloy was by about 12%. The hardness of E3 alloys was above 100 HBW, which are very high values for aluminum alloys cast in ceramic molds. In the case of alloys F1 to F3, the HBW hardness values reached 90 to 98. In the evaluation of HBW, an increase was recorded in all experimental melts of F1 to F3 up to about 10% compared to R alloys. As can be seen (Figure 14), alloys E1 to E3 show a significant increase in HBW. 

## 4. Discussion

The change of the segregation temperatures for the structural components and shape of curves was mainly associated with the influence of Ti and Zr in the investigated alloy [38]. Ti acted as an element that promoted the formation of nuclei for the future Zr phases. This fact was also confirmed in the evaluation of microstructures. An increased number of precipitated Zr phases was observed in alloys E1 to E3 compared to alloy P. In the alloy alloyed with Zr and Sr, the increase in phase precipitation temperatures compared to the R and P alloy represented about 30 °C. This increase is probably related to the influence of Sr as an element (modifier) acting on the transformation of the eutectic. However, no significant change in the cooling temperature was observed for the Zr phases, which was related to the segregation of Zr phases in a similar amount as for the P alloy.

The gas content rate determined by the Dichte Index for alloys E1 to E3 was increased by 77% compared to the R alloy. The effect on hydrogen bubble entrapment may be related to the shape, size, or number of formed Zr phases. These can act as “absorbers” of hydrogen bubbles. Zr phases are formed in the investigated alloy in acicular morphology at a temperature of about 630 °C, while the resulting nuclei can trap the formed hydrogen bubbles. By adding Ti at a Zr content of 0.15 wt. % (alloys E1–E3) there was an increase in gas content compared to the R alloy. As presented in the chapter Evaluation of Microstructure, in experimental variants of Zr with Ti, the number of formed Zr phases increased with increasing Ti content in alloys E1 to E3. Although experimental melts with a combination of Zr and Ti did not precipitate Zr to the same extent (dimensions) as the P alloy, the increased number of forming Zr phases may prevent hydrogen bubbles from “escaping” from the melt. The reduction in the size of the formed Zr phases does not cause a significant increase in DI (1) compared to the P alloy. 

As with P and the E1 to E3 alloys, the effect of formed Zr phases could cause hydrogen bubbles to be trapped during solidification. The corresponding increase in DI is associated not only with the synergistic effect of Zr and the formation of Zr-dispersoids, but also with the higher content of Sr, which at higher additions forms undesirable oxides such as SrO and Al_2_SrO_3_.

The effect of Ti as a nucleating element of Zr phases in the experimental alloy was confirmed in samples E1 to E3. Based on the evaluation of mechanical properties, the optimal Ti content was determined to be 0.2 wt. % with an interaction of 0.15 wt. % Zr. At this content, an increased content of segregated Zr phases with a balanced ratio of smaller, sharp-ended needles and square-shaped phases was recorded in the structure of the E2 alloy. An increased number of skeletal structures in the matrix of experimental alloys was also observed, while the interaction of phases based on Mg, Cu, and Fe with Zr phases also occurred. A similar phenomenon was observed for P alloy samples. This fact confirms the significant ability of Zr phases to strengthen the metal matrix and thus increase the hardness of the investigated alloys. It is assumed that these phases will also have a stabilizing effect and allow use even at higher temperatures (above 250 °C). The evaluation of the structure confirmed the corrective effect of Zr as an element that eliminates long acicular phases of Fe and creates a more favorable morphology. Due to the elimination of an increased number of Zr phases, it was possible to observe a decrease in the ductile failure of the matrix when evaluating the fracture surfaces of experimental alloys E1 to E3.

Increasing the B content by adding the AlTi5B master alloy to alloys E1–E3 was not demonstrated in the chemical composition evaluation (Table 1), and the B content was not identified. The absence of TiB_2_ phases could cause a high Si content. Together with Ti, they form silicides (TiSi, TiSi_2_, or Ti_5_Si_3_) which exhaust the possibilities of TiB_2_ formation [39].

Experimental alloys with Zr and Sr were characterized by the precipitation of Zr phases, in a similar morphology to that observed for the P alloy. With a Sr content above 0.2 wt. % there was an increase in the interaction between intermetallic phases based on Cu, Mg, and Fe. This fact confirms the positive effect of the synergy between Zr and Sr, with the possibility of increasing the strength of the metal matrix of the alloy AlSi7Mg0.3Cu0.5. Due to the increased Sr content (premodification), the formation of brittle phases of the AlSr_2_Si_2_ type could occur, which, together with the formation of Zr intermetallic phases with sharp-edged ends, multiplied the transcrystalline cleavage and thus significantly affected the reduction in A_5_. At the same time, the increased content of brittle phases based on AlSr_2_Si_2_ promoted an increase in porosity. Alloys of variant F, with the exception of the F2 alloy, achieved slightly higher values of A_5_ than the R alloy.

When comparing the mechanical characteristics of the experimental variants E1–E3 and F1–F3 with the P and R alloy, the highest mechanical characteristics were obtained for the alloys E1 and E2. The highest values of R_m_ and R_p0.2_ were achieved by alloy F2. Due to the synergistic effect of Zr and Ti in the investigated alloys, there was an increase in R_m_ and R_p0.2_ compared to P and R alloys. From the given results, it can be stated that the alloy with added Zr and Sr achieved better mechanical properties than the AlSi7Mg0.3Cu0.5 alloyed with Zr with Ti. Due to the segregation of Zr phases on a much larger scale, material variants with the addition of Zr and Sr achieve low ductility A_5_ values. Unfortunately, A_5_ values are very low for almost all alloys. The achieved ductility is unacceptable, e.g., for functional engine components. 

The highest values of the modulus of elasticity were measured for material variants with the addition of Zr and Ti-alloy E3 (97 GPA). It can be concluded that the obtained values of the modulus of elasticity reached very high values in almost all investigated alloys. The hypothesis was confirmed that the synergistic effect of Zr and Ti and Zr and Sr can significantly increase the stress resistance, also with respect to the formation of stable Zr-based dispersoids in the matrix. The above facts give preconditions for the use of these alloys for castings requiring higher heat resistance and heat strength. In alloys E1 to E3 and F3, Brinell hardness reached very high values. This fact may be related to a larger number of smaller Zr phases formed in samples E1 and E3, which have a reinforcing character.

## 5. Conclusions

The results from individual experimental material variants can be summarized in the following conclusions:

- The addition of alloying elements Zr (P-alloy), Zr and Ti (E1–E3), and Zr and Sr (F1–F3) to the AlSi7Mg0.3Cu0.5 alloy causes an increase in gas content. The highest gas content is caused by the synergistic effect of Zr and Sr.
- The synergistic effect of Zr and Ti, especially Ti < 0.2 wt. % with constant addition of Zr 0.15 wt. % (alloys F1–F3), affects the formation of an increased number of Zr phases with smaller dimensions. Zr phases are formed in an angular form.
- Effect of Zr and Sr at constant addition of Zr 0.15 wt. % (alloys F1–F3) on the investigated alloy is manifested by the formation of Zr phases with an angular end and a position concentrated mainly in the eutectic domain.
- The monitored mechanical characteristics of HBW and the modulus of elasticity have reached high values, which gives a precondition for the use of given materials for demanding castings operating at elevated temperatures.

The expected problem is the low values of A_5_ ductility, which will still need to be worked on.

In conclusion, it can be stated that the effect of alloying elements manifested positively, either individually or synergistically on the AlSi7Mg0.3Cu0.5 alloy. In the current state of knowledge, the highest mechanical characteristics were achieved by alloys E2 (addition of 0.12 wt. %. Zr and 0.2 wt. % Ti).

## Figures and Tables

**Table 1:** Chemical composition of reference, primary, and experimental alloys (wt. %).

Alloy	Si	Zr	Ti	Sr	Fe	Cu	Mn	Mg	B
**R—AlSi7Mg0.3Cu0.5**	6.88	-	0.10	0.01	0.12	0.54	0.07	0.37	<0.000
**P—AlSi7Mg0.3Cu0.5Zr0.15**	6.86	0.13	0.16	0.01	0.12	0.55	0.07	0.37	<0.000
**E1—AlSi7Mg0.3Cu0.5Zr0.15Ti0.1**	6.96	0.12	0.23	0.01	0.13	0.55	0.07	0.37	<0.000
**E2—AlSi7Mg0.3Cu0.5Zr0.15Ti0.2**	6.68	0.14	0.28	0.01	0.13	0.54	0.07	0.35	<0.000
**E3—AlSi7Mg0.3Cu0.5Zr0.15Ti0.3**	6.51	0.13	0.37	0.01	0.13	0.52	0.07	0.34	<0.000
**F1—AlSi7Mg0.3Cu0.5Zr0.15Sr0.1**	6.55	0.13	0.11	0.10	0.11	0.51	0.07	0.36	<0.000
**F2—AlSi7Mg0.3Cu0.5Zr0.15 Sr0.2**	6.38	0.13	0.10	0.19	0.11	0.50	0.07	0.35	<0.000
**F3—AlSi7Mg0.3Cu0.5Zr0.15 Sr0.3**	6.04	0.14	0.10	0.29	0.11	0.5	0.07	0.35	<0.000

**Table 2:** Material composition of ceramic mold layers.

Component	1. Layer	2. Layer	3. Layer	4. Layer	5. Layer
**Binder**	Primcot cote plus	SP-Ultra 2408	MatriXsol 30	MatriXsol 30	MatriXsol 30
**Grain**	Cerabed DS 60	Rancosil A	Molochite 30–80 DD	Molochite 30–80 DD	Molochite 30–80 DD

**Table 3:** Temperatures of structural components formation in R, P, and experimental melts E1–E3.

Variant	R Alloy	P Alloy	E1 Alloy	E2 Alloy	E3 Alloy
**α phase (°C)**	616	619	617	623	613
**Eutectic (°C)**	565	563	556	565	560
**Zr-rich phase (°C)**	-	630	639	645	640

**Table 4:** Temperatures of structural components formation in R, P, and experimental melts F1–F3.

Variant	R Alloy	P Alloy	F1 Alloy	F2 Alloy	F3 Alloy
**α phase (°C)**	616	619	613	629	624
**Eutectic (°C)**	565	563	580	591	589
**Zr–rich phase (°C)**	-	630	635	636	633

## Data Availability

Data available on request. The data presented in this study are available on request from the corresponding author.
